# Photolithographic Patterning of Cytop with Limited Contact Angle Degradation

**DOI:** 10.3390/mi9100509

**Published:** 2018-10-09

**Authors:** Yalei Qiu, Shu Yang, Kuang Sheng

**Affiliations:** College of Electrical Engineering, Zhejiang University, Hangzhou 310027, China; 3110103374@zju.edu.cn (Y.Q.); eesyang@zju.edu.cn (S.Y.)

**Keywords:** microfluidics, electrowetting-on-dielectric (EWOD), Cytop, patterning, annealing

## Abstract

Cytop is a commercially available amorphous fluoropolymer with excellent characteristics including electric insulation, water and oil repellency, chemical resistance, and moisture-proof property, making it an attractive material as hydrophobic layers in electrowetting-on-dielectric (EWOD) devices. However, its highly hydrophobic surface makes it difficult for photoresists to be directly coated on the surface. To pattern Cytop, plasma treatment prior to applying photoresists is required to promote the adhesion between the photoresist and the Cytop coating. This approach inevitably causes hydrophobicity loss in the final EWOD devices. Thus, a damage-reduced recipe for Cytop patterning is urgently needed. In this paper, we first characterized the damage caused by two categories of surface treatment methods: plasma treatment and metal treatment. Parameters such as plasma gas source (Ar/O_2_), plasma treatment time (0–600 s), metal target (Al/Cu/Cr/Au), metal deposition process (magnetron sputtering or e-beam evaporation) were varied. Film thickness, wettability, and roughness were quantified by ellipsometry measurements, contact angle measurements, and atom force microscope (AFM), respectively. We then evaluated the effectiveness of annealing in damage reduction. Experimental results show that: (1) annealing is necessary in restoring hydrophobicity as well as smoothing surfaces; (2) specified film thickness can be obtained by controlling plasma treatment time; (3) “Ar/O_2_ plasma treatment + an AZ5214 soft mask + annealing” is a feasible recipe; (4) “an Al/Cu/Cr/Au hard mask + annealing” is feasible as well.

## 1. Introduction

Microfluidics is currently an active research field in both academia and industry. Among various technologies to control fluid behavior in micro scale [1], digital microfluidics based on electrowetting-on-dielectric (EWOD) phenomenon stands out [2,3,4], by virtue of its digital control scheme and simple device structure, making it an attractive candidate for system integration with other components of lab-on-chip (LOC). A basic EWOD device consists of three layers: a conduction layer, a dielectric layer, and a hydrophobic layer. The conduction layer is used to apply electric field for droplet actuation. Conductor materials such as Au, Cr, Al, or indium tin oxide (ITO) can be deposited and patterned to form contact pads, interconnects, as well as actuation electrodes. The dielectric layer is used to withstand the intensity of electric field between the conduction layer and the conducting fluid, therefore avoiding electrolysis. High-k dielectrics such as SiN_x_, Al_2_O_3_, or HfO_2_ are preferred in low-voltage EWOD devices. The hydrophobic layer is used to increase the modulation range and decrease the contact angle (CA) hysteresis of EWOD actuation. Teflon, FluoroPel, and Cytop are three common choices as hydrophobic layer materials. In 2013, CJ Kim’s group did a thorough evaluation of repeated electrowetting on these three fluoropolymers [5]. EWOD samples with the same configuration of a 50 nm hydrophobic layer coated on top of a 560 nm SiO_2_ dielectric layer on a Si substrate are fabricated using varied coating products (0.78% Teflon AF 1600 from DuPont, Wilmington, DE, USA, 1% FluoroPel 1601V from Cytonix, Beltsville, MD, USA, or 1.7% Cytop CTL-809M from Asahi Glass, Tokyo, Japan). EWOD performances are then tested under three types of repeated actuation conditions (positive DC, negative DC, or 1 kHz AC) using contact angle range (∆CA) reduction as a degradation indicator in a modified sessile drop test setup. Experimental results showed that under the tested conditions: (1) for DC actuations, electrowetting degrades gradually on Cytop but significantly faster on Teflon and FluoroPel; (2) for AC actuations, electrowetting degrades gradually on all three materials in a similar fashion. Consequently, Cytop provides superior overall performance to the other two.

In their 2013 paper [6], Chae et al. investigated the relationship between Cytop film thickness and its surface wettability in order to identify the optimum thickness for operating voltage reduction in EWOD systems. For Cytop films prepared directly by spin-coating, sessile drop tests showed that the minimum thickness to maintain a static contact angle of approximately 110° is 3 nm. Furthermore, tilting plate tests indicated that to obtain a low contact angle hysteresis of approximately 4° film thickness shall be at least 12 nm. Nevertheless, scarce information can be found in literature concerning Cytop patterning in EWOD devices, which is a pivotal technological barrier for EWOD devices (as actuators) to be integrated with other components of LOC, especially sensors. To our knowledge, the common practice [7,8] to keep contact pads uncovered by Cytop is by applying low tack tapes to the protected regions before Cytop solution deposition and removing them after Cytop curing. This technique is handy when fabricating small-volume samples, yet unscalable and incompatible with general-purpose cleanroom processes. According to the technical document [9] provided by the manufacturer (AGC Chemicals Fluoroproducts Division, Asahi Glass, Tokyo, Japan), a recommended recipe for Cytop pattern processing starts with surface modification by plasma treatment for ease of resist application, then followed by photolithography and dry etching. However, this approach inevitably causes hydrophobicity loss in the final EWOD devices. So, a natural question arises: how to keep the water-repelling performance not degraded during the lithography process? In 2017, Nardecchia et al. adopted a lift-off process to pattern Cytop in an effort to integrate capillary and EWOD technologies in micro-analytical systems [10]. Although patterning is achieved without sacrificing hydrophobicity, the quality of a lift-off process relies heavily on the anisotropic deposition profile of the film. In case of Cytop, solution deposition method results in poor pattern resolution. Thus, a scalable, damage-reduced, high-resolution recipe for Cytop patterning in EWOD devices is urgently needed.

In order to develop a feasible recipe that meets the above requirements, a comprehensive literature review on Cytop processing was performed. It turns out that apart from being extensively used as hydrophobic coatings in EWOD devices, Cytop is also used as bridge dielectrics in GaAs-based enhancement-mode p-HEMTs [11], as gate dielectrics in organic small molecule FETs [12], as encapsulation materials in organic LEDs [13], and as cladding materials in planar polymer waveguides [14], among which the last work caught our attention. In this 2012 work, Leosson et al. overcame the difficulty of securing adhesion between layers in fabricating Cytop-PMMA-Cytop multilayer photonic structures, paving the way for Cytop to be used in planar polymer waveguides for biophotonics. They found that for Cytop CTX-809AP2, several minutes in Ar plasma can reduce the contact angle to below 60°, however, such plasma treatment did not ensure sufficient adhesion to subsequent layers. The solution they ended up with to ensure both wettability improvement and good adhesion consists of a pretreatment by vacuum deposition of a thin layer (10 nm or more) of Al on the Cytop surface and subsequent removal by etching in an alkaline solution (NaOH). The contact angle was reduced to 83° after such metal treatment, which provided us with a promising new direction parallel to the conventional method of plasma treatment. In their earlier 2010 work [15], they observed that after Al treatment, heating to above 100 °C restores the original hydrophobicity of the Cytop surface, which indicates that the influence exerted by Al treatment is reversible in nature. Further reviewing the technical document [16], we learned that Cytop has a low glass transition temperature (T_g_ = 108 °C). Pulling all this information together, we inferred that a low-temperature annealing process might be helpful for reducing the film damage introduced by surface treatments.

**Figure 1:**
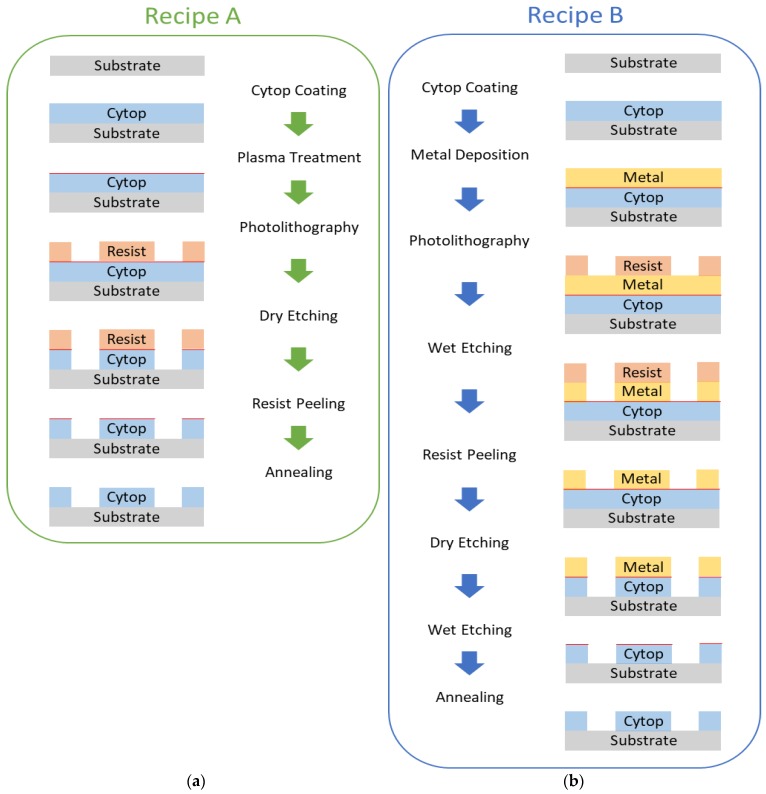
Process flow for the two developed recipes: (**a**) Recipe A includes 6 steps in total. The first step is “Cytop Coating”. Substrates are cleaned and Cytop films are prepared on top of the substrates by spin-coating. Then, “Plasma Treatment” is used to promote the adhesion of photoresists on the Cytop surface. The red line represents the damage to the Cytop surface during plasma treatment. Then during “Photolithography”, photoresists are patterned to serve as the soft mask for Cytop patterning. Then “Dry Etching” is performed to transfer the pattern from the soft mask to the Cytop film. Then “Resist Peeling” is followed to remove the soft mask. At last, “Annealing” is required to eliminate the damage from the finished Cytop pattern; (**b**) Recipe B includes 8 steps in total. After “Cytop Coating”, the second step is “Metal Deposition”. Metal films can be directly deposited on top of Cytop films. Again, the red line represents the damage to the Cytop surface during the deposition process. Then, “Photolithography” is used to fabricate a photoresist mask for metal patterning, and “Wet Etching” is performed to transfer the pattern from the photoresist mask to the metal film, and “Resist Peeling” is followed to finish the hard mask. Then “Dry Etching” is performed to transfer the pattern from the hard mask to the Cytop film. Then “Wet Etching” is followed to remove the hard mask. At last, again “Annealing” is required to eliminate the damage from the finished Cytop pattern.

In this paper, generally two kinds of recipes are investigated and validated: one using a soft mask, the other one using a hard mask. Process flow for the two developed recipes are shown in Figure 1. Critical process parameters are characterized and validated through controlled experiments (see Section 2 and Section 3). In recipe A, plasma treatment prior to applying photoresists is required to promote the adhesion between the photoresist and the Cytop coating. The soft mask is fabricated by photolithography and Cytop pattern is dry-etched. Annealing after photoresist removal is required to counterbalance the damage (the red line in Figure 1a) caused by plasma treatment. In recipe B, a metal film is directly deposited on top of Cytop, then patterned by wet etching to serve as the hard mask for Cytop patterning. Likewise, annealing is necessary to counterbalance the damage (the red line in Figure 1b) caused by metal treatment.

## 2. Experiments

### 2.1. Experiment Design

In search of a feasible recipe that is scalable, high-resolution as well as damage-reduced, our basic idea is to adopt the framework of the manufacturer’s recipe [9], which satisfies the first two requirements, and adapt it to meet the third requirement. Drawing inspirations from the literature review, we aspire to minimize damage-introduction or maximize damage-elimination by experimentally weighing the two options (plasma treatment & metal treatment) from two perspectives: (1) effectiveness for surface modification; (2) reversibility under post-treatment (annealing).

Surface modification of fluoropolymers, such as poly (tetrafluoroethylene) (PTFE), perfluoro alkoxy copolymer (PFA), and poly (vinyl fluoride) (PVF), via chemical treatment or plasma treatment [17], has been widely studied. Chemical treatment of fluoropolymers has been shown to be an effective method to achieve surface defluorination and refunctionalization. Depending on the type of reactive etchants used, various extents of defluorination and the incorporation of new functional groups, such as oxygen-, sulfur-, and silicon-containing groups, have been observed. Despite their effectiveness in activating and refunctionalizing the fluoropolymer surfaces, chemical treatments share one limitation in that the depth profile of the modification is difficult to monitor and control. The bulk properties of the fluoropolymers are inevitably affected by the reactive etchants during chemical treatment, which introduces pervasive, irreversible damage. Comparatively, plasma treatment of fluoropolymers is surface-sensitive: plasma affects the polymer surface to an extent of several hundred to several thousand angstroms. The bulk properties of fluoropolymers, therefore, remain unchanged. Glow discharge generated from various gases has been used to modify the surface composition of fluoropolymers in similar ways as chemical treatment. Defluorination and incorporation of new functional species, such as the oxygen- and nitrogen-containing species, are generally observed after plasma treatment, which introduces surface-confined, irreversible damage.

**Figure 2:**
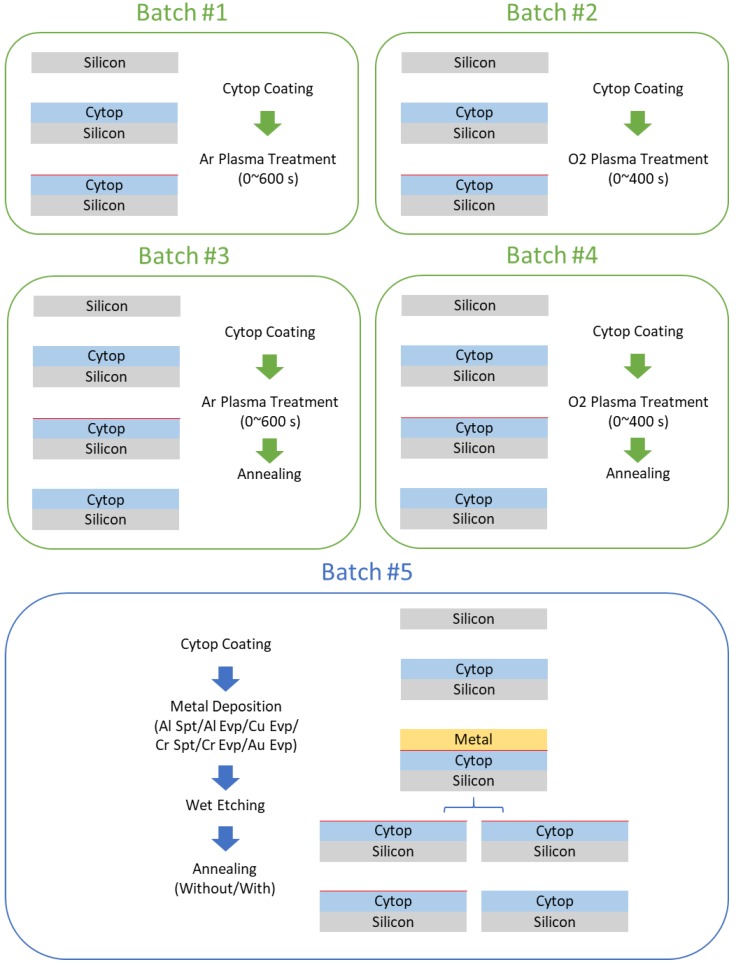
Experiment design.

In our controlled experiment, we designed five batches of samples in total, and deliberately set the treatment conditions for each batch (experiment design shown in Figure 2). For the sake of processing convenience, batch #1 and batch #2 were separated to evaluate the effectiveness of plasma treatment in surface modification while batch #3 and batch #4 were complemented to evaluate the reversibility of plasma treatment under annealing. For the sake of rigorous comparison, batch #5 was designed to incorporate effectiveness evaluation and reversibility evaluation of metal treatment into one batch. Both effectiveness evaluation and reversibility evaluation were implemented through quantitative characterization of the Cytop film. We decided to choose film thickness, wettability, and roughness as the three indicators for film damage characterization because these three indexes are critical for device performance. During our evaluations, we first characterized the damage caused by plasma treatment and metal treatment. Critical process parameters of plasma treatment, including plasma gas source (Ar/O_2_) and plasma treatment time (0–600 s), were varied. Likewise, for metal treatment, parameters such as metal target (Al/Cu/Cr/Au), metal deposition process including magnetron sputtering (abbr. Spt) or e-beam evaporation (abbr. Evp) were varied. We then evaluated the effectiveness of annealing in damage reduction.

### 2.2. Experiment Materials

The controlled experiment itself requires only two kinds of materials: Cytop films as the subject under investigation, and silicon wafers as the substrate. Cytop solution CTL-809M and solvent CT-SOLV180 were purchased from Asahi Glass Co., Ltd. (Tokyo, Japan). CTL-809M is a special type of Cytop solution with 9% monomer concentration (standard-grade) and –CONH-SiOR end-groups (M-grade). M-grade was chosen because it provides direct adhesion to inorganic substrates without silane pretreatment, and it is suitable for EWOD applications. Silicon wafers (2-inch, single-sided polishing) were purchased from Zhejiang Lijing Photoelectric Technology Co., Ltd. (Quzhou, China) with consideration of easy handling. For further complete verification of the proposed recipes, boro-aluminosilicate glass slides (25 mm × 50 mm × 1.1 mm) were purchased from Delta Technologies Co., Ltd. (Loveland, CO, USA) to replace silicon wafers as the substrate since transparency is generally preferred in EWOD devices.

### 2.3. Experiment Procedures

The first stage of the experiment was Cytop film preparation. Silicon wafers were successively immersed in acetone, isopropanol, and absolute ethanol, and ultrasonically cleaned for 15 min at 25 °C. After organic cleaning, silicon wafers were rinsed by deionized water and blown dry using N_2_ gas. Cytop solution CTL-809M (monomer concentration 9%) was diluted to CTL-805M (monomer concentration 5%) before spin-coating. The spin cycle involved a 600 rpm spread for 5 s and a 3000 rpm spin for 50 s. After spin-coating, the samples were left to dry at room temperature for 5 min. Then, the samples were cured on a hotplate at 80 °C for 0.5 h and subsequently at 185 °C for 1 h. After curing, the samples were cooled till room temperature in air.

The second stage of the experiment was Cytop surface treatment and annealing. Plasma treatment was carried out using 50 sccm Ar or O_2_ at 80 mTorr and 50 W RF power using a plasma asher (AP-1000, Nordson MARCH, Concord, CO, USA). Metal deposition was carried out by depositing 25 nm Al or Cu or Cr or Au at 5 × 10^−6^ Torr and room temperature using magnetron sputtering (PVD 75, Kurt J. Lesker Company, Jefferson Hills, PA, USA) or e-beam evaporation (Cooke Vacuum Products, South Norwalk, CT, USA). Wet etching was carried out by using corresponding etchants to etch away the metal films. Al, Cu, Cr, Au films were etched using commercial aluminum etching solution, homemade 30% (wt.) FeCl_3_ saturated solution, commercial chromium etching solution, homemade gold etching solution (400 g I_2_:100 g KI:400 mL H_2_O), respectively. The condition for annealing was 230 °C for 1 h on a hotplate.

The final stage of the experiment was Cytop film characterization. Film thickness, wettability, and roughness were quantified by ellipsometry measurements (M-2000, J.A. Woollam, Lincoln, CA, USA), contact angle measurements (OCA 20, Dataphysics Instruments, Filderstadt, Germany), and atom force microscope (MultiMode, Veeco Instruments, Plainview, NY, USA), respectively. Thickness values were fitted using the Cauchy model. Contact angles values were averages of 5 drops of ultrapure water, each 3.5 microliters in volume, placed in various positions across the sample surface. Roughness values were averages of 3 probed square areas, each 10 micrometers in side length, across the sample surface.

## 3. Results

**Figure 3:**
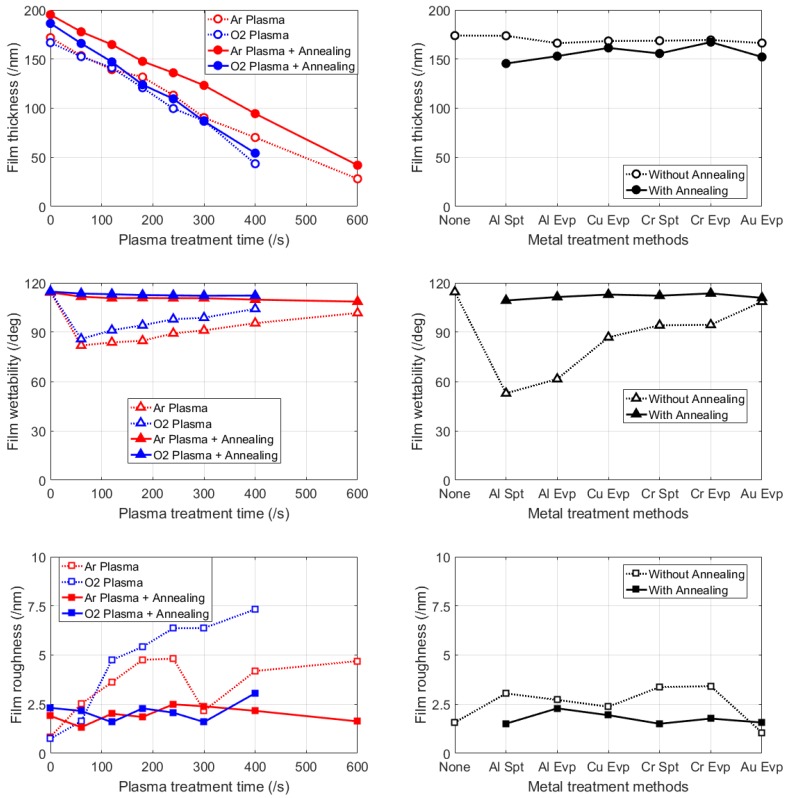
Experimental results. The upper-left chart shows the relationships between “Film thickness” and “Plasma treatment time” under four combinations of treatment conditions: “Ar Plasma” alone, “O_2_ Plasma” alone, “Ar Plasma” plus “Annealing”, “O_2_ Plasma” plus “Annealing”. The middle-left chart shows the relationships between “Film wettability” and “Plasma treatment time” under the same conditions. The lower-left chart shows the relationships between “Film roughness” and “Plasma treatment time” under the same conditions. The upper-right chart shows the relationships between “Film thickness” and “Metal treatment methods” under two conditions: without “Annealing”, with “Annealing”. The middle-right chart shows the relationships between “Film wettability” and “Metal treatment methods” under the same conditions. The lower-right chart shows the relationships between “Film roughness” and “Metal treatment methods” under the same conditions.

Experimental data are collected, processed, and plotted in Figure 3, which comprises 6 charts in total. All the thickness data (round mark) from batch #1 to batch #5 were gathered into the charts on the first row. Likewise, all the contact angle data (triangular mark) were gathered into the middle row, and all the roughness data (square mark) were gathered into the last row. Further, data from batch #1 to batch #4 were synthesized into the left column for better illustration of the effectiveness (hollow mark) and reversibility (solid mark) of plasma treatment (red & blue mark). Likewise, data from batch #5 were placed into the right column to illustrate the effectiveness and reversibility of metal treatment (black mark).

## 4. Discussion

### 4.1. The Effects of Plasma Treatment and Annealing

Both Ar plasma and O_2_ plasma treatment exert a linear etching effect on Cytop films, while O_2_ plasma has a slightly faster etching speed (0.304 nm/s) over Ar plasma (0.242 nm/s). To our understanding, such difference is due to different dominant etching mechanisms between O_2_ and Ar, namely chemical reaction and physical bombardment, respectively. The linear relationship can be utilized to obtain the target film thickness through control of plasma treatment time, especially in low-voltage electrowetting where precise control of hydrophobic layer thickness in the tens of nanometers range brings significant performance enhancements [18]. After annealing, the slope of the fitting line “thickness vs. time” increases slightly for both O_2_ plasma (from 0.304 nm/s to 0.329 nm/s) and Ar plasma (from 0.242 nm/s to 0.252 nm/s).

In terms of modification of Cytop surface wettability, Ar plasma and O_2_ plasma treatment result in similar curves with a concave shape: a brief time of exposure effectively renders Cytop surfaces hydrophilic, whereas longer exposure time leads to a steady increase in hydrophobicity. This tendency is consistent with the findings from [15] where Cytop CTX-809AP2 surfaces were exposed to plasmas of O_2_, O_2_/CHF_3_, and O_2_/Ar in various compositions. Although Ar has a slightly stronger influence on wettability than O_2_, further spin-coating experiments revealed that all the plasma-treated samples show good adhesion to photoresists AZ5214E, indicating large process windows (Ar, 60–600 s and O_2_, 60–400 s) for adhesion promotion. With annealing, surface hydrophobicity is virtually restored by promoting atomic migration [19].

Despite the benefits of thickness control and adhesion promotion, a side effect of plasma treatment is surface roughening. Obviously, under O_2_ plasma treatment, Cytop surface roughness shows a positive correlation with plasma treatment time. In contrast, under Ar plasma treatment, Cytop surface roughness shows a nonmonotonic trend with extended plasma treatment time. This fluctuation characteristic of Ar plasma, whether periodical or not, is beneficial to restrain surface roughness within a certain range. Nevertheless, it is only after annealing that surface roughening is effectively eliminated.

### 4.2. The Effects of Metal Treatment and Annealing

Compared with plasma treatment, metal treatment barely etches Cytop films. This is because neither the metal deposition process nor the subsequent wet-etching process is destructive for the stable fluoropolymer structure. However, all metal treatment methods examined (except Au evaporation) significantly modify surface wettability, among which Al treatment is the most effective method to reduce the contact angle (in particular, Al sputtering reduces the contact angle to below 60°). According to the mechanism explained in [14], upon Al deposition, acid-base interactions occur between acidic (electron-accepting) aluminol sites and basic (electron-donating) carbonyl functional groups. Additionally, ionic bonding may occur between the carboxylate anion of the Cytop end group and aluminum, causing the end groups of the polymer to reorient to the polymer–aluminum interface. Upon removal of the aluminum layer by wet etching in an alkaline solution, the carbonyl groups remain oriented to the Cytop-air interface, reducing the hydrophobicity of the surface. Another observation is that the hydrophobicity reduction effect of the metal treatments is strongly dependent on the metal types (Al > Cu > Cr > Au), but less dependent on the deposition processes, which indicates that different metal materials may have different degrees of acid-base interactions and ionic bonding. Furthermore, such correlation between the metal treatment and the resulting surface wettability suggests an alternative way besides plasma treatment for adhesion enhancement before photoresists application. At last but not the least, the surface roughening effect of the metal treatments is relatively modest.

With annealing, samples processed by different metal treatment methods undergo different degrees of thinning effect. More importantly, annealing cancels out the effects of the metal treatments: restoring hydrophobicity as well as smoothing surfaces.

## 5. Conclusions

Based on the characterization results, we have successfully completed the validation of the critical process steps of the two proposed recipes. We have found that: (1) Cytop surface modification through Ar/O_2_ plasma treatment exerts three major effects: linear etching, adhesion promotion, and surface roughening; (2) Cytop surface modification through Al/Cu/Cr metal treatment exerts two major effects: adhesion promotion and surface roughening; (3) Au metal treatment barely modifies Cytop films; (4) Cytop surface modification either through Ar/O_2_ plasma treatment or through Al/Cu/Cr metal treatment possesses excellent reversibility under annealing, that is, hydrophobicity restoring and surface smoothing; (5) annealing is generally necessary after Cytop surface treatments for damage reduction, except for Au metal treatment.

To further verify the feasibilities as well as qualities of the two proposed recipes, we have fabricated samples with Cytop test patterns on glass slides. Microscope inspection of the samples shows that we have successfully developed two scalable, high-resolution, damage-reduced recipes for Cytop patterning in EWOD devices.
